# Genetic Disruption of *Toxoplasma gondii* *peroxiredoxin* (*TgPrx*) 1 and 3 Reveals the Essential Role of *TgPrx3* in Protecting Mice from Fatal Consequences of Toxoplasmosis

**DOI:** 10.3390/ijms23063076

**Published:** 2022-03-12

**Authors:** Ragab M. Fereig, Yoshifumi Nishikawa

**Affiliations:** 1 National Research Center for Protozoan Diseases, Obihiro University of Agriculture and Veterinary Medicine, Inada-cho, Obihiro 080-8555, Hokkaido, Japan; ragabfereig2018@gmail.com; 2 Department of Animal Medicine, Faculty of Veterinary Medicine, South Valley University, Qena City 83523, Qena, Egypt

**Keywords:** *Toxoplasma gondii*, peroxiredoxin, toxoplasmosis, virulence

## Abstract

*Toxoplasma gondii* is a worldwide protozoan parasite that endangers human health and causes enormous economic losses to the animal production sector. A safe and effective vaccine or treatment is needed to reduce these hazards. In this study, we revealed the cyto-nuclear and mitochondrial localization of TgPrx1 and TgPrx3 proteins, respectively. We knocked out the *T. gondii peroxiredoxin* (*TgPrx*KO) 1 and 3 genes using a parental type II Prugniaud strain lacking KU80 and HXGPRT genes (PruΔku80Δhxgprt) via CRISPR-Cas9 technology. The successful KO was confirmed using PCR, IFAT, and Western blotting in two clones of both target genes, named *TgPrx1*KO and *TgPrx3*KO. Regarding *in vitro* assays, no significant variations between any of the knocked-out clones in *TgPrx1*KO or *TgPrx3*KO parasite strains, or even PruΔku80Δhxgprt, were obtained in rates of infection, proliferation, or egress. Nevertheless, mice that were infected with tachyzoites of the *TgPrx3*KO strain showed a marked decrease in survival rate compared with *TgPrx1*KO- and PruΔku80Δhxgprt-infected mice. This effect was confirmed using different mouse strains (ICR and C57BL/6J mice), sexes (male and female), and immunological backgrounds (ICR and SCID mice). In addition, *TgPrx1*KO and *TgPrx3*KO induced high levels of interferon gamma (IFN-γ) in infected mice at 8 days post infection, and increased IL-6 and IL-12p40 production from murine macrophages cultivated *in vitro*. The results of the present study suggested that *TgPrx3* can induce anti-*T. gondii* immune responses that protect the mice from fatal consequences of toxoplasmosis. The results of our current and previous studies represent *TgPrx3* as an excellent candidate for sub-unit vaccines, suggesting it may contribute to the control of toxoplasmosis for susceptible humans and animals.

## 1. Introduction

As a member of the phylum Apicomplexa, *Toxoplasma gondii* (*T. gondii*) is an obligate intracellular protozoan parasite that can infect almost all warm-blooded animals such as humans and cats. It is estimated that about one third of the world’s human population has been infected with *T. gondii* [1,2]. Although the infection is usually asymptomatic under normal conditions, it can cause severe complications and even death in immunocompromised individuals. In addition, toxoplasmosis has a severe economic impact on the sheep and goat industries because it induces abortion, stillbirth, and neonatal losses. However, there are no successful *Toxoplasma* vaccines that can be applied clinically [2].

Most of the previous studies focused on rhoptries, micronemes, dense particles, and secretory proteins as virulence or immune effector molecules. However, there has been no significant breakthrough in current research regarding new virulence factors [3]. *T. gondii* possesses a highly functional antioxidant system including a group of enzymes such as glutathione peroxidase, superoxide dismutase, catalase, and peroxiredoxins (Prxs), which perform their role independently or synergistically to protect the parasite against adverse conditions *in vivo* or *ex vivo* [4]. To date, three *Prx* genes have already been identified and characterized functionally and biochemically. Based on the number of active redox cysteine residues and the localization in the cell, the Prxs classified as 1-cys-Prx (TgPrx1) and 2-cys-Prx (TgPrx2) were identified in the cytoplasm of the parasite, while 2-cys-Prx (TgPrx3) was expressed in the mitochondrion, either in tachyzoite or bradyzoite stages [4]. Peroxiredoxin-linked detoxification of reactive oxygen species plays a critical role in protecting *T. gondii* against oxidative stress during its life cycle. This effect makes the peroxiredoxins promising drug targets or vaccine candidates against *T. gondii* [5,6,7].

Few reports have investigated the immunogenicity of TgPrxs. A previous study assessed the immunomodulatory role of TgPrx1 derived from the RH strain using an *in vitro* model only. The recombinant TgPrx1 enhanced the activity of alternatively activated macrophages (AAM), triggering the production of IL-10 and high expression of arginase-1. Simultaneously, rTgPrx1 induced downregulation of IL-1β secretion from AAM [8]. Consistently, our previous reports [9,10] provided more inclusive data regarding the immunomodulatory role of the recombinant protein of TgPrx1 and TgPrx3. Both rTgPrx1 and rTgPrx3 were investigated as vaccine candidates, and they demonstrated significant protective immunity in immunized mice compared with control non-immunized groups. This protection was accomplished by triggering T-helper 1 and 2 immune responses [9,10]. These results prompted us to further assess TgPrx1 and TgPrx3 as vaccine candidates, drug targets, or virulence factors, using knockout techniques as the more recent and reliable approach.

To explore the molecular function of the *TgPrx1* and 3 genes, we generated *TgPrx1*- or *TgPrx3*-deficient lines of *T. gondii* type II PruΔku80Δhxgprt (*TgPrx1*KO and *TgPrx3*KO), using clustered regularly interspaced short palindromic repeats (CRISPR)-associated protein 9 (CRISPR/Cas9). Our results suggest that the phenotype of *TgPrx*3KO plays a key role in modulating the host’s immunity toward the anti-*Toxoplasma* activity.

## 2. Results

### 2.1. Localization of TgPrx1 and TgPrx3

**Figure 1:**
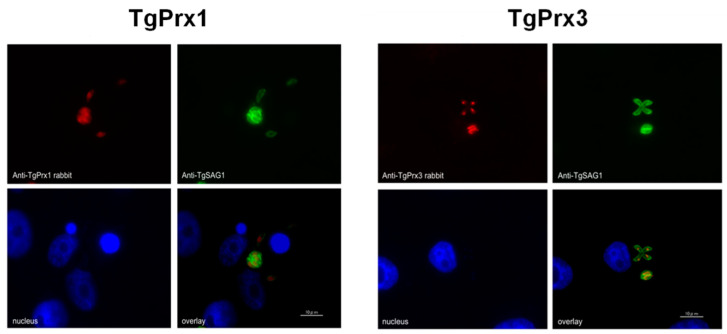
Localization of TgPrx1 and TgPrx3. IFAT analysis of Vero cells infected with PruΔku80Δhxgprt parasites at 48 h post infection. Expression of TgPrx1 was visualized with anti-TgPrx1 rabbit serum (red) and mouse TgSAG1 monoclonal antibodies (green). Expression of TgPrx3 was visualized with anti-TgPrx3 rabbit serum (red) and mouse TgSAG1 monoclonal antibodies (green). Overlay indicates localization of TgPrx1 or TgPrx3 with TgSAG1 proteins. The nuclear DNA was stained with Hoechst stain (blue). Scale bar: 10 μm.

To determine the cellular localization of TgPrx1 and TgPrx3, *T. gondii*-infected Vero cells were stained via specific anti-sera against TgPrx1 and TgPrx3 prepared in BALB/c mice. A monoclonal antibody against TgSAG1 was also used as a control antigen to stain the surface of tachyzoites. The IFAT results showed that TgPrx1 was expressed in the cytoplasm and nucleus (cyto-nuclear expression), while TgPrx3 was located in the mitochondrion of *T. gondii* (Figure 1).

### 2.2. Construction of TgPrx1KO and TgPrx3KO Parasites

**Figure 2:**
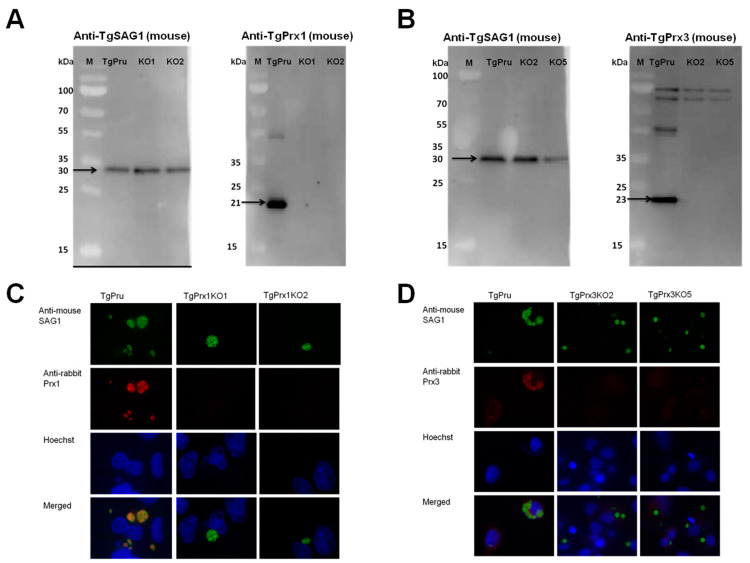
Generation of *TgPrx1*KO and *TgPrx3*KO parasites using CRISPR/Cas9. Western blot analyses of *TgPrx1*KO and *TgPrx3*KO parasites. TgSAG1 and TgPrx1 were detected using monoclonal mouse anti-SAG1 and polyclonal anti-TgPrx1 mouse serum. Black arrow indicates target band of TgSAG1 (30 kDa) and TgPrx1 (21 kDa). TgPru; PruΔku80Δhxgprt, KO1: *TgPrx1*-deficient parasite clone 1 (*TgPrx1*KO1); KO2: *TgPrx1*-deficient parasite clone 2 (*TgPrx1*KO2); M: marker (Panel **A**). TgSAG1 and TgPrx3 were detected using monoclonal mouse anti-SAG1 and polyclonal anti-TgPrx3 mouse serum, respectively. Black arrow indicates target band of TgSAG1 (30 kDa) and TgPrx3 (23 kDa). TgPru; PruΔku80Δhxgprt, KO2: *TgPrx3*-deficient parasite clone 2 (*TgPrx3*KO2); KO5: *TgPrx3*-deficient parasite clone 5 (*TgPrx3*KO5); M: marker (Panel **B**). Characterization of *TgPrx1*KO parasites by IFAT (Panel **C**,**D**). Vero cells were infected with PruΔku80Δhxgprt, *TgPrx1*-deficient parasite clone 1 (*TgPrx1*KO1), *TgPrx1*-deficient parasite clone 2 (*TgPrx1*KO2), *TgPrx3*-deficient parasite clone 2 (*TgPrx3*KO2) or *TgPrx3*-deficient parasite clone 5 (*TgPrx3*KO5) for 48 h. Expression of TgPrx1 and TgPrx3 was visualized with anti-TgPrx1 and TgPrx3 rabbit serum (red), respectively, and mouse anti-TgSAG1 monoclonal antibody (green) as a control antibody. The nuclear DNA was stained with Hoechst stain (blue).

The CRISPR-Cas9 technique was used to disrupt the *TgPrx1* and *TgPrx3* genes in type II PruΔku80Δhxgprt (Figure 2A). Single clones obtained from drug selection and limiting dilution methods were first confirmed by PCR to exclude parental parasites. To verify the successful establishment of the gene-deficient lines, PCR was used to confirm the insertion of the DHFR* cassette into the target gene in the clones obtained with limited dilution. The amplification of the target gene was negative, and the insertion of the DHFR* cassette into the target gene was confirmed in each deficient line (2 KO clones in *TgPrx1* and 2 KO clones in *TgPrx3*) (see Figures S1 and S2 in the Supplementary Material). For further confirmation, the loss of the target genes was also confirmed with Western blotting (Figure 2). Anti-TgPrx1 mouse serum detected a 21 kDa protein in the PruΔku80Δhxgprt strain, but not in TgPrx1KO clones 1 and 2 (Figure 2A). Similarly, anti-TgPrx3 mouse serum detected a 23 kDa protein in the PruΔku80Δhxgprt strain, but not in *TgPrx3*KO clones 2 and 5 (Figure 2B). Simultaneously, the control anti-TgSAG1 mouse monoclonal antibody detected the expected size band of 30 kDa for protein in all the lysates of parental and KO parasites. In addition, we confirmed the loss of target protein expression by IFAT in the tested KO clones of *TgPrx1* (clone 1 and 2) and *TgPrx3* (clone 2 and 5). In addition, TgSAG1 was expressed in the parental and all KO parasites (Figure 2C,D).

### 2.3. Effect of TgPrx1 or TgPrx3 Gene Disruption on the Growth and Egress of T. gondii

**Figure 3:**
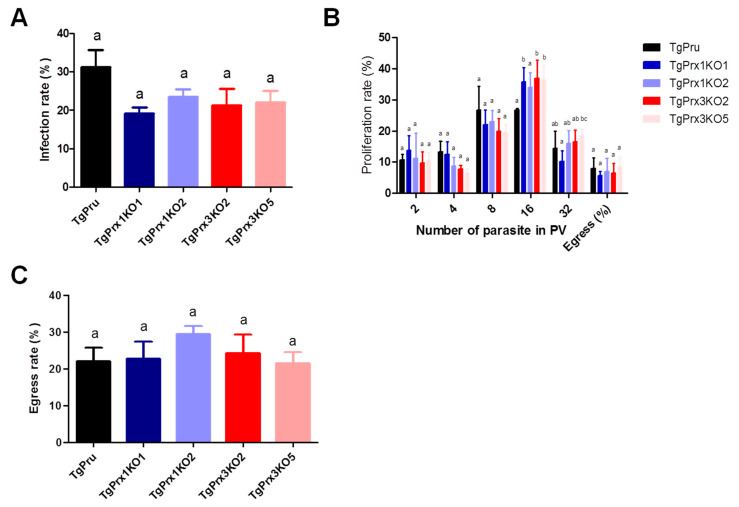
*In vitro* infection, proliferation, and egress rate. (**A**) Infection rates of the different parasite lines in Vero cells at 24 h post infection. (**B**) Intracellular replication assay of the parasite lines in Vero cells at 48 h post infection. (**C**) Egress rates of the different parasite lines in Vero cells at 72 h post infection. Each bar represents the means and standard deviations (n = 3 for all groups). Different letters above the bars indicate the statistically significant differences among the groups, according to a one-way ANOVA (**A**,**C**) or two-way ANOVA (**B**) and a Tukey–Kramer post hoc analysis (*p* < 0.05). TgPru refers to PruΔku80Δhxgprt. *TgPrx1*KO1 is *TgPrx1*-deficient parasite clone 1, *TgPrx1*KO2 is *TgPrx1*-deficient parasite clone 2, *TgPrx3*KO2 is *TgPrx3*-deficient parasite clone 2, and *TgPrx3*KO5 is *TgPrx3*-deficient parasite clone 5.

A growth kinetic assay was used to compare the characteristics of *TgPrx1* or *TgPrx3* knockout strains and parental PruΔku80Δhxgprt strains (Figure 3). Vero cells grown in a 12-well plate were infected with tachyzoites of *TgPrx* mutants or parental strains for analyzing infection (24 hpi), proliferation (48 hpi), or egress rates (72 hpi). Two clones from *TgPrx1*KO and *TgPrx3*KO were used in these experiments for better assessment. The results showed that there were no significant differences in the infection or egress rates between cells infected with any clone from *TgPrx1*KO and *TgPrx3*KO tachyzoites and those of parental strains. Regarding the proliferation rate, the size of vacuoles also showed no differences in all test strains in vacuoles containing 2-8 parasites. On the contrary, the number of vacuoles containing 16 parasites in *TgPrx1*KO2- and *TgPrx3*KO2- or *TgPrx3*KO5-infected cells was significantly higher than in parental PruΔku80Δhxgprt-strain infected cells. In addition, the number of vacuoles containing 32 parasites in *TgPrx3*KO5-infected cells was higher than in *TgPrx1*KO1 only (Figure 3). In general, these results demonstrate that the disruption of *TgPrx1* or *TgPrx3* genes did not hamper the growth or the infection of the parasite *in vitro*.

### 2.4. Pathogenicity of Parental and Knockout Strains of TgPrx

**Figure 4:**
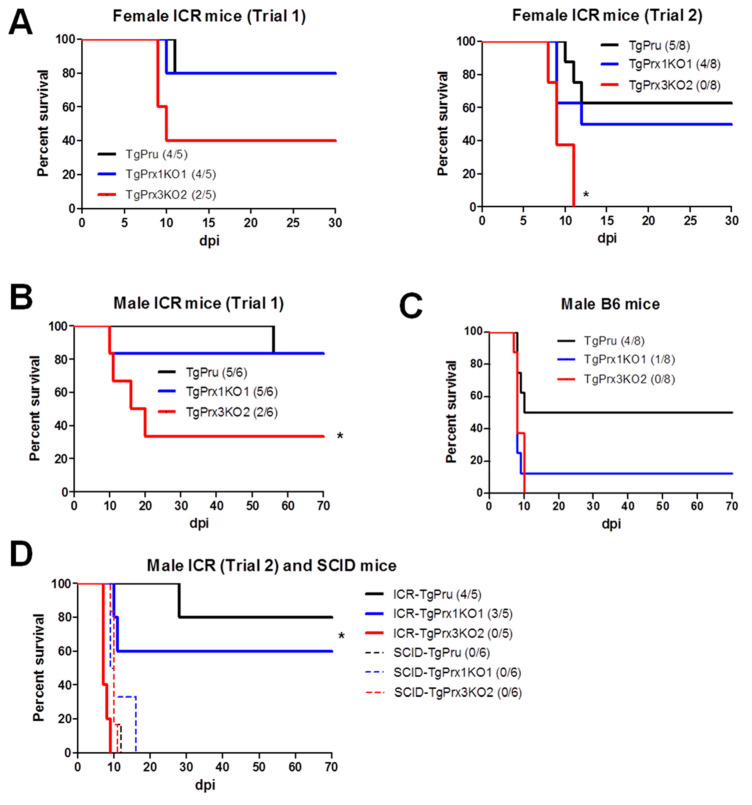
Survival rates in different mouse strains. (**A**) Female ICR mice were infected intraperitoneally with (5 × 10^4^) tachyzoites of PruΔku80Δhxgprt (TgPru), *TgPrx1*-deficient parasite clone 1 (*TgPrx1*KO1) or *TgPrx3*-deficient parasite clone 2 (*TgPrx3*KO2) (*n* = 5 for trial 1 and *n* = 8 for trial 2). Male ICR (**B**) and male C57BL/6 (**C**) mice were infected intraperitoneally with (5 × 10^4^) tachyzoites of different parasite lines (*n* = 6 for ICR and *n* = 8 for C57BL/6 mice). Survival was monitored twice daily until 30 and 70 dpi in female and male mice experiments, respectively. (**D**) Male ICR and SCID mice were infected intraperitoneally with 5 × 10^4^ tachyzoites of TgPru, *TgPrx1*KO1, or *TgPrx3*KO2 (*n* = 5 for trial 2 of male ICR, and *n* = 6 for SCID). Survival was monitored twice daily until 70 dpi. Asterisk (*) refers to a significant difference among the test groups. Statistical analysis was performed using a log rank test (*p <* 0.05).

Different mouse strains were used to assess the survival rate after infection with different parasite strains via an intraperitoneal route. In the case of ICR mice, two trials were conducted in both male and female mice. In the case of female ICR mice, the survival rate was greatly reduced in *TgPrx3*KO2-infected mice compared with those infected with *TgPrx1*KO1 and parental parasites: *TgPrx3*KO2 (2/5, 40%), *TgPrx1*KO1 (4/5, 80%), and PruΔku80Δhxgprt (4/5, 80%) in trial 1, and *TgPrx3*KO2 (0/8), *TgPrx1*KO1 (4/8, 50%), and PruΔku80Δhxgprt (5/8, 62.5%) in trial 2. However, the difference was statistically significant in trial 2 but not in trial 1 (*p* < 0.05) (Figure 4A). In addition, male ICR and C57BL/6 mice were infected with tachyzoites of PruΔku80Δhxgprt, *TgPrx1*KO1, or *TgPrx3*KO2 to confirm the virulence. Significant differences among the *TgPrx3*KO2-infected group (2/6, 33.3%) and other test groups (PruΔku80Δhxgprt (5/6, 83.3%) and *TgPrx1*KO1 (5/6, 83.3%)) were obtained in the case of ICR mice (*p* < 0.05) but not in C57BL/6 mice, although a similar effect was reported (*TgPrx3*KO2 0/8; *TgPrx1*KO1 1/8, 12.5%; PruΔku80Δhxgprt 4/8; 50%) (Figure 4B,C). In the same context, another experiment was performed including male ICR mice (trial 2) and SCID mice to investigate the role of lymphocytes in the immunoprotective effect of *TgPrx3*. SCID mice lacking B- and T-lymphocytes showed higher vulnerability to all parasite strains, and all mice in the different groups (six mice per group) succumbed during the acute and sub-acute stages of infection (16 dpi). In contrast, male ICR mice showed higher mortality in *TgPrx3*KO-infected mice (0/5) than in PruΔku80Δhxgprt- (4/5, 80%) and *TgPrx1*KO-infected mice (3/5, 60%) (Figure 4D). This result indicated that *TgPrx3*KO increased the virulence of parasites in different mouse models and showed the possible role of lymphocytes in the induced protective immunity against the parental strain of *T. gondii*.

The clinical findings, parasite burden, and antibody response were also investigated in female ICR mice. Mice infected with *TgPrx3*KO2 tachyzoites showed a higher clinical score and decreased body weight compared with those infected with PruΔku80Δhxgprt and *TgPrx1*KO1 parasites, despite the short course of infection. Showing a similar tendency, the parasite burden in the brains of *TgPrx3*KO2-infected mice was higher than in those infected with *TgPrx3*KO1 or PruΔku80Δhxgprt, although this was not statistically significant. However, the antibody levels against TgGRA7 were similar in all mouse groups infected with different parasite lines and only showed significantly higher levels against the non-infected mouse group (*p* < 0.05) (Figure S3).

### 2.5. In Vivo and In Vitro Cytokine Production

**Figure 5:**
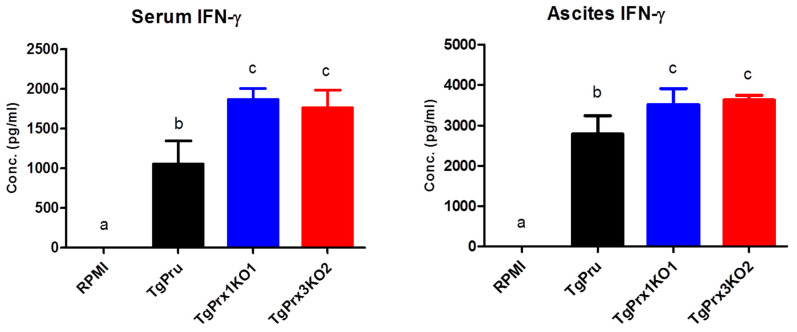
*In vivo* production of IFN-γ. Female ICR mice (*n* = 4/group) were infected intraperitoneally with (5 × 10^4^) tachyzoites via PruΔku80Δhxgprt (TgPru), *TgPrx1*-deficient parasite clone 1 (*TgPrx1*KO1) or *TgPrx3*-deficient parasite clone 2 (*TgPrx3*KO2) or inoculated with RPMI only as a control non-infected group. At day 8 after infection, serum and ascitic fluid were collected to detect IFN-γ. The different letters above the bars in the graphs indicate statistically significant differences among the different groups using one-way ANOVA followed by a Tukey–Kramer post hoc analysis (*p <* 0.05).

To unravel the mechanism of the induced high virulence of *TgPrx3*KO2 compared with PruΔku80Δhxgprt and *TgPrx1*KO1 parasites, the cytokines levels were determined using *in vivo* and *ex vivo* experiments. At day 8 after infection, serum and ascitic fluid were collected from female ICR mice infected with different test parasites, to detect IFN-γ as a marker of sickness in mice. Significantly higher levels of IFN-γ were recorded in both *TgPrx1*KO- and *TgPrx3*KO-infected mice than in PruΔku80Δhxgprt-infected mice (*p* < 0.05), in both serum and ascites samples (Figure 5).

**Figure 6:**
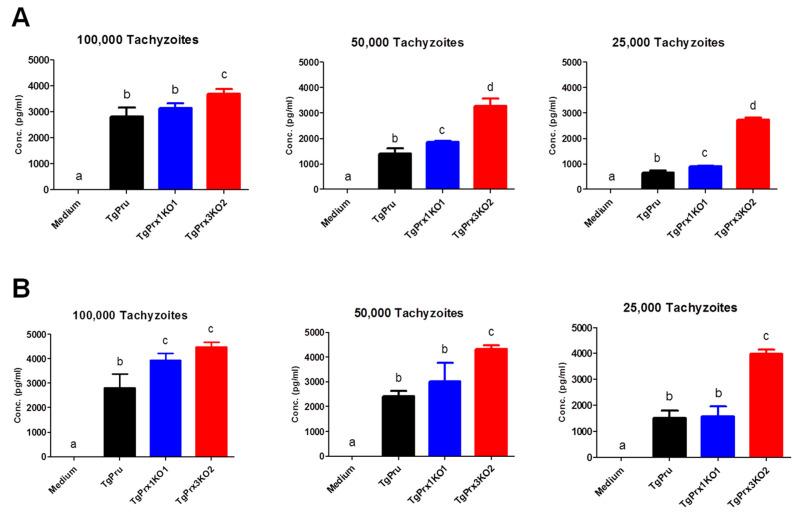
*In vitro* effect of *TgPrx1* and 3 disruptions on IL-12p40 (**A**) and IL-6 (**B**) production by mouse peritoneal macrophages. Macrophages were infected with different numbers of tachyzoites from the parental strain PruΔku80Δhxgprt (TgPru) or the knockout strains *TgPrx1*-deficient parasite clone 1 (*TgPrx1*KO1) or *TgPrx3*-deficient parasite clone 2 (*TgPrx3*KO2) or only treated with medium for 20 h. The IL-12p40 (**A**) and IL-6 (**B**) levels were measured in the culture supernatant. Each value represents the mean ± standard deviation of quadruple samples. The reproducibility of the data was confirmed with two independent experiments. The different letters above the bars in the graphs indicate statistically significant differences among the different test groups (one-way ANOVA plus Tukey–Kramer post hoc analysis, *p* < 0.05).

In addition, murine peritoneal macrophages were infected *in vitro* with different numbers of PruΔku80Δhxgprt, *TgPrx1*KO, and *TgPrx3*KO. Then, the IL-12p40 and IL-6 levels were measured in the culture supernatant. Higher levels of IL-12p40 and IL-6 were recorded in *TgPrx3*KO-infected cell supernatant than in PruΔku80Δhxgprt- or *TgPrx1*KO-infected cell supernatants (*p* < 0.05). This effect was highly remarkable at low infective doses (50 and 25 × 10^3^) compared with a higher dose (100 × 10^3^) of parasites (Figure 6). These results suggested the greater ability of the *TgPrx3*KO parasite to induce cytokine production *in vivo* and *in vitro* compared with PruΔku80Δhxgprt and both PruΔku80Δhxgprt and *TgPrx1*KO parasites, respectively.

## 3. Discussion

To date, three *TgPrx* genes have been identified in the *T. gondii* genome (*TgPrx1*, *TgPrx2*, and *TgPrx3*). The biochemical characterization of *TgPrx*s regarding antioxidant enzymes was the most extensively investigated role of such genes [4,6,11]. However, few reports have demonstrated the immunomodulatory effects of TgPrxs including TgPrx1 [8,10] and TgPrx3 [9]. It is noteworthy that most of the above-mentioned reports relied on using recombinant proteins of TgPrx in the biochemical or immunological assessments. The current study provides the first report of the investigation of *TgPrx1* and *TgPrx3* (2-cys peroxiredoxin) using gene deletion or the disruption approach. In the same context, only one previous study has succeeded in deleting *TgPrx2* (1-cys peroxiredoxin) and has also investigated its role as antioxidant enzyme [12].

Assessment of the protein functions in parasites is primarily based on gene editing or protein expression analyses. Recently, the application of the CRISPR/Cas9 technology has led to great advancements in efficient gene manipulation in apicomplexan parasites including *T. gondii* [12]. The CRISPR/Cas9 system was adapted to produce efficient targeted gene disruption and the site-specific insertions of selectable markers in *T. gondii* [13,14]. Recently, we succeeded in investigating the role of TgGRA7 and TgGRA14 using CRISPR/Cas9, with a similar approach to that applied in the current study [15]. This study showed that TgGRA7 and TgGRA14 induced host immunity via NFκB modulation.

Most previous studies indicated that TgPrx1 was localized in the cytoplasm, while TgPrx3 expression was observed in the mitochondrion [4,5,6]. However, a more recent study referred to a controversial issue by showing evidence of nuclear localization of TgPrx1 in addition to cytosolic expression, using the same antibody [7]. Data obtained in our study also indicated the cytosolic and nuclear expression of TgPrx1.

In this study, we generated *TgPrx1-* and *TgPrx3*-deficient *T. gondii* PruΔku80Δhxgprt strains to examine the roles of the encoded proteins. The characterization of the specified *TgPrx* in *T. gondii* was confirmed by PCR, Western blotting, and IFAT. Two clones from both *TgPrx1*KO and *TgPrx3*KO parasites were used to confirm the stability of the obtained data. Correct and precise insertion of the DHFR* cassette was confirmed using different primers to amplify specific sequences from *TgPrx* and the inserted construct. Western blotting revealed the absence of TgPrx1 and TgPrx3 protein in KO parasites, while it was clearly observed at the expected size in parental strains. In addition, our IFAT results showed the specific localization of TgPrx1 (cyto-nuclear) and TgPrx3 (mitochondrial) in the parental parasites but not the KO parasites. These results indicated the successful gene disruption of both *TgPrx1* and *TgPrx3*.

Based on previous reports, the Prxs genes were expected to be potential drug targets, because of their critical role as antioxidant enzymes that protect the parasites against deleterious oxidative stressors [5,16]. Indeed, Conoidin A can bind covalently to the peroxidatic cysteine of TgPrx2, inhibiting its enzymatic activity *in vitro*. However, disruption of the *TgPrx2* gene by homologous recombination had no effect on the sensitivity of the parasites to Conoidin A, suggesting that TgPrx2 is not the invasion-relevant target of this compound [17]. Accordingly, we sought to check the candidacy of TgPrx1 and TgPrx3, as drug targets and for the discovery of specific inhibitors. However, the disruption of both *TgPrx1* and *TgPrx3* did not affect the abilities of the parasite with regard to infection, replication, or egress *in vitro* using Vero cells. In this experiment, two clones from each KO parasite (*TgPrx1*KO and *TgPrx3*KO) were used to check the data stability. Furthermore, the mice infection experiments abolished our strategy to check TgPrx1 and TgPrx3 as drug targets due to the increased or similar virulence against the parental parasite in *TgPrx3*KO and *TgPrx1*KO, respectively. This result was confirmed using various mouse models including different mouse strains and sexes.

However, many previous reports demonstrated the similar phenomenon of increased parasite virulence after deletion of certain genes. Deletion of TgGRA7, TgGRA14, and TgGRA15 were demonstrated to increase the virulence of the parasites, in the case of either type I RHΔku80Δhxgprt or type II PruΔku80Δhxgprt strains [15]. Similarly, mice infected with TgGRA15KO parasites showed a higher parasite burden and higher growth rate *in vitro* than those infected with the parental parasite [18]. Furthermore, a deficiency of aspartate aminotransferases in both RH and PLK did not reduce the virulence in mice, although the growth ability of the parasites was affected *in vitro* [19]. This phenomenon demonstrates the close co-evolution of *T. gondii* and host. *T. gondii* evolved in ways that preserved the host’s wellbeing up to a point, avoiding extreme damage to the host and ensuring parasite survival and continuation of the life cycle.

*Toxoplasma gondii* has a fascinating ability to manipulate the effector immune cells and molecules to maintain its survival and to keep the host alive. Both cellular and humoral immunity is involved in combating the parasite. Macrophages are the first line of host defense against many infectious agents including *T. gondii.* They are responsible for the production of a vast group of cytokines that are essential for regulating the immune response against invading *T. gondii*. In the early stage of infection, classically activated macrophages can produce a group of proinflammatory cytokines such as interleukin 6 (IL-6), IL-12, and tumor necrosis factor alpha (TNF-α). In addition, IL-12 can promote the function of natural killer cells and T-cells to produce interferon gamma (IFN-γ). These cytokines can act synergistically to mediate the killing of the parasite by macrophages [20,21,22].

The increased virulence of the *TgPrx3*KO parasite in mice may suggest the immunoprotective role of such antigens during infection. However, the deletion of the *TgPrx1* gene did not affect the parasite phenotype either *in vitro* or *in vivo*, as found for *TgPrx3* in the current study. In fact, both TgPrx1 and TgPrx3 are 2-cys peroxiredoxins, but they have different localizations of expression, so various effects might be expected.

To identify the mechanism of the protective role of TgPrx3 during infection, the IFN-γ level was measured as a marker of sickness in serum and ascites of mice at 8 dpi, which was the day on which severe infection was observed. We found that mice infected with both *TgPrx3*KO and *TgPrx1*KO parasites showed higher IFN-γ secretion than those infected with the parental PruΔku80Δhxgprt strain. However, in another experiment, higher levels of proinflammatory cytokines IL-6 and IL-12p40 were observed mainly in *TgPrx3*KO parasites against both *TgPrx1*KO- and PruΔku80Δhxgprt-infected macrophages. These results may explain the high severity of infection in the case of *TgPrx3*KO-infected mice. Interestingly, this result was different from those we obtained previously, as both recombinant TgPrx1 [10] and rTgPrx3 [9] triggered the IL-12 production from macrophages. However, this different effect might be correlated with the different approaches of KO and recombinant protein assays. In the case of parasite infection, there is an intricate effect of numerous protein–protein interactions, even for the parasite itself, rather than using one antigen as in the recombinant protein assessment approach. However, our previous studies reported that the use of rTgPrx1 and rTgPrx3 as vaccine antigens promoted cellular and humoral immune responses and improved mouse survival [9,10]. These results corroborate the findings of the current study concerning the efficient role of TgPrx1 and TgPrx3 in manipulating host immunity towards protecting the host against the damaging effect of the parasite. Another aspect of the induced high virulence of parasites lacking *TgPrx3* may be related to its potential role as an antioxidant enzyme. This antioxidant effect was not investigated in the current study, but it is the most frequently investigated aspect in all identified Prxs of *T. gondii* including *TgPrx3* [4,6,16]. Oxidative stress caused by accumulation of reactive oxygen species (ROS) such as hydrogen peroxide and nitric oxide has a highly harmful effect on the host cells either *in vivo* or *in vitro*. The infection with *T. gondii* resulted in the production of high levels of a variety of ROS triggered by the activation of macrophages or other immune cells [23,24].

In this study, we confirmed the successful knockout of *TgPrx1* and *TgPrx3* using different experimental approaches (PCR, Western blotting, and IFAT). In addition, two clones from each KO parasite were used in the experiments to corroborate the obtained data and the effect of gene deletion. However, our efforts to generate complementary parasites for *TgPrx1*KO or *TgPrx3*KO were not successful, suggesting further attempts to investigate the role of *TgPrx1* and *TgPrx3* are necessary, using a similar approach. These findings can be exploited in the further assessment of TgPrx3 as a vaccine antigen against *T. gondii* using various animal models and different vaccination strategies.

## 4. Materials and Methods

### 4.1. Ethics Statement

The current study was conducted strictly according to the recommendations of the Guide for the Care and Use of Laboratory Animals of the Ministry of Education, Culture, Sports, Science and Technology, Japan. The protocol was approved by the Committee on the Ethics of Animal Experiments at Obihiro University of Agriculture and Veterinary Medicine, Obihiro, Japan (permit numbers 20-22, 19-50, 18-38, 29-39). All painful experiments were conducted under isoflurane anesthesia, and every effort was made to minimize animal suffering.

### 4.2. Animals

Female and male ICR, male C57BL/6 and SCID mice, and female BALB/c mice, at 6 to 8 weeks of age, were obtained from Clea Japan (Tokyo, Japan). These mice were housed under specific-pathogen-free conditions in cages in the animal facility of the National Research Center for Protozoan Diseases at Obihiro University of Agriculture and Veterinary Medicine, Obihiro, Japan. The animals used in this study were treated and used according to the Guiding Principles for the Care and Use of Research Animals published by the Obihiro University of Agriculture and Veterinary Medicine.

### 4.3. Parasite and Cell Cultures

All the parasite strains used in this study (*T. gondii* type II PruΔku80Δhxgprt, *TgPrx1*KO, and *TgPrx3*KO) were maintained in African green monkey kidney epithelial cells (Vero cells) cultured in Eagle’s minimum essential medium (Sigma, St. Louis, MO, USA) containing 8% heat-inactivated fetal bovine serum (FBS) in a 37 °C and 5% CO_2_ incubator. *T. gondii* type II PruΔku80Δhxgprt was kindly gifted by Professor David Bzik (Dartmouth Medical School). Prior to each experiment, the parasites were purified from host cell debris and washed with cold phosphate-buffered saline (PBS). Finally, the pellet was resuspended carefully in Roswell Park Memorial Institute (RPMI) 1640 medium (Sigma, St. Louis, MO, USA) and passed through a 27-gauge needle and a 5.0 µm pore size filter (Millipore, Bedford, MA, USA).

### 4.4. Plasmid Construction and Generation of Knockout Parasites

The *TgPrx1* and *TgPrx3* gene knockout strains were constructed as described in the literature, using CRISPR-Cas9 [13,15,25]. SgRNA from *TgPrx1* and *TgPrx3* was briefly transmitted into pSAG1:CAS9-U6:sgUPRT by PCR using a Q5 Mutagenesis Kit, and positive plasmid of pSAG1:CAS9-U6:sg *TgPrx1* or *TgPrx3* was extracted using Endo-Free Plasmid DNA Mini Kit protocols (Takara, Kusatsu, Shiga, Japan). The dihydrofolate reductase (DHFR) resistance cassettes were amplified from the plasmid pUPRT-DHFR-D (Addgene, Watertown, MA, USA) by PCR reaction and purified by agarose gel electrophoresis. About 50 μg of positive plasmids and 5 μg of purified DHFR amplicons were cotransfected into freshly harvested PruΔku80Δhxgprt tachyzoites by electroporation, as described previously [25]. Stably resistant clones were selected by growth in pyrimethamine (1 µM) for 14 days and were subsequently screened via PCR to ensure the correct integration of the DHFR cassette into each target gene locus (see Figures S1 and S2 and Table S1 in the Supplementary Material. The PCR-positive clones were further analyzed via Western blotting and the indirect fluorescent antibody test (IFAT) to confirm the loss of the target gene. Two clones from each parasite were identified and confirmed, named *TgPrx1*KO1, *TgPrx1*KO2, *TgPrx3*KO2, and *TgPrx3*KO5.

All the plasmids and primers used in this study are listed in Table 1 and Table S1. Further details of the plasmid construction can be found in the Supplementary Methods in the Supplementary Material.

### 4.5. Expression and Purification of Recombinant Proteins

The *TgPrx1* and *TgPrx3* genes were amplified from cDNA of the *T*. *gondii* PLK strain, as described in our previous studies [9,10]. In brief, PCR products digested with Bam*HI* and *Xho*I were inserted into the pGEX-4T3 plasmid vector (Table S1). Recombinant TgPrx1 and TgPrx3 were expressed as glutathione *S*-transferase (GST) fusion protein in *Escherichia coli* BL21(DE3) (New England Biolabs Inc., Ipswich, MA, USA).

### 4.6. Preparation of Lysate Antigen

Lysate antigen from purified tachyzoites of *T. gondii* (PruΔku80Δhxgprt), *TgPrx1*KO, or *TgPrx3*KO was prepared as previously reported [10]. The obtained extract was measured using a BCA protein assay kit to detect the concentrations (Thermo Fisher Scientific, Waltham, MA, USA).

### 4.7. Western Blot Analysis

The protein lysates from purified *T*. *gondii* tachyzoites (2 or 5 μg/10 μL for *TgPrx1*KO and *TgPrx3*KO, respectively) were mixed with 10 μL of 2×SDS reducing gel-loading buffer (62.5 mM Tris-HCl pH 6.8, 2% (*w*/*v*) SDS, 140 mM 2-mercaptoethanol, 10% (*w*/*v*) glycerol and 0.02% (*w*/*v*) bromophenol blue). Other procedures were applied as described in our previous report [10]. Briefly, samples were heated at 95°C for 5 min and separated on a 15% polyacrylamide gel. The membranes were incubated with mouse anti-TgSAG1 (monoclonal antibody F77D; Waltham, MA, USA), or anti-TgPrx1 [10] or anti-TgPrx3 [9] mouse serum (1:200) for 1 h at room temperature. After washing three times, the membranes were incubated with anti-mouse horseradish peroxidase-conjugated immunoglobulin G (1:2000; Amersham Pharmacia Biotech, Piscataway, NJ, USA) diluted in PBS–skim milk (3%), for 1 h at 37 °C. The protein bands were visualized using ECL™ Western blotting detection reagents (GE Healthcare UK Ltd., Buckinghamshire, UK) using a VersaDoc™ imaging system (Nippon Bio-Rad Laboratories, Tokyo, Japan) according to the manufacturer’s recommendations.

### 4.8. Indirect Fluorescent Antibody Test (IFAT)

A Vero cells suspension of 5 × 10^4^ cells in 1 mL of MEM supplemented with 8% FBS was seeded in each well in a 12-well plate with a coverslip and incubated at 37 °C in a 5% CO_2_ atmosphere for 24 h. The cells were infected with purified tachyzoites of different parasite lines at 2 × 10^5^/1 mL of MEM and incubated again at 37 °C in a 5% CO_2_ atmosphere for 2 h, followed by washing with PBS to remove dead parasites. Plates were incubated again under the same conditions for 48 h for characterization experiments, 24 h for infection rate, 48 h for proliferation rate, and 72 h for egress rate assessment [15,25]. Old medium was aspirated, and coverslips were washed with PBS 3 times, followed by fixation with 3% paraformaldehyde in PBS (*v/v*) for 30 min at RT. After washing 2 more times with PBS, permeation of cells was achieved by adding 0.3% Triton X-100 in PBS and keeping for 5 min at RT. The coverslips were washed 3 more times followed by blocking with 3% bovine serum albumin (BSA) in PBS (BSA–PBS) for 30 min at RT. As for the first antibodies, cells were stained by fluorescent-labeled antibodies; anti-mouse TgSAG1 monoclonal antibodies (Invitrogen) (1:500) or anti-rabbit TgPrx1 [10] or TgPrx3 [9] polyclonal antibodies (1:200). For secondary anti-mouse or anti-rabbit antibodies, Alexa Fluor (1:500 dilution, Sigma, St. Louis, MO, USA) and Hoechst (1:1000, Thermo Fisher Scientific Inc., Waltham, MA, USA) were used as green or red staining, depending on the type of antibodies used, diluted 1:500 in 3% BSA–PBS for 1 h at RT. The coverslips were placed on a glass slide containing a fresh drop of Mowiol (Calbiochem, San Diego, CA, USA), and the slides kept in the dark for at least 2 h. Slides were examined using an all-in-one fluorescence microscope (BZ-9000, Keyence, Tokyo, Japan).

### 4.9. Infection, Growth, and Egress Rates of T. gondii Lines

Vero cells were plated at 1 mL/well in a 12-well plate with a coverslip by suspension, at 5 × 10^4^ in MEM 8% FBS, then incubated for 20 h at 37 °C. Parasites were plated in at 1 × 10^5^ in MEM using 1 mL/well (multiplicity of infection 1:4 host to parasite cells). The parasites that failed to invade cells were washed away, and the cultures were incubated again as previously mentioned. Counting, calculation, and analyses were performed as described previously [15]. After 24 h, 48 h, or 72 h, the cells were fixed by paraformaldehyde (3%) in PBS and prepared for IFAT for detection of infection, proliferation, or egress rates, respectively. The infection rates were calculated using IFAT as follows: (number of TgSAG1-positive Vero cells/100 randomly selected Vero cells) × 100. To measure the *T. gondii* proliferation rate in the Vero cells, the sizes of the parasitophorous vacuoles (PVs) were determined by counting the number of parasites per PV (in a total of 100 randomly selected vacuoles) and expressed as a percentage (%) of the total PV based on the TgSAG1 fluorescent signals. In the case of egress rate, i.e., parasite egress in the Vero cells, the percentage of egressed vacuoles was calculated by scoring at least 100 vacuoles as intracellular or egressed, relying on TgSAG1 fluorescent signals. All precautions were applied to avoid any errors and duplicate counting. Additionally, all assessment procedures for infection, replication, or egress experiments were conducted blindly by a neutral person by hiding the slide ID.

### 4.10. In Vivo Assessment of PruΔku80Δhxgprt and TgPrxKO Strains in Mice

To compare the virulence of *T. gondii* in mice, female and male ICR, male C57BL/6, and male SCID mice were intraperitoneally injected with different parasite lines of *T. gondii* (5 × 10^4^ tachyzoites/mouse). The mouse survival was assessed in all trials of different mice (30 days post infection (dpi) in female and 70 dpi in male mice). In addition, body weight and clinical score were monitored daily for up to 30 dpi, and the sera and brains of female ICR mice were collected at the sacrifice time of 30 dpi. To analyze cytokine production *in vivo*, sera and ascites were collected from the female mice infected with PruΔku80Δhxgprt, *TgPrx*1KO1, or *TgPrx*3KO2 strains or inoculated with RPMI medium only (*n* = 4 per group) at 8 dpi.

### 4.11. Monolayer Cultures of Peritoneal Macrophages

Peritoneal macrophages from female ICR mice were collected 4 days after the intraperitoneal injection of 1 mL of 4.05% Brewer’s modified thioglycollate medium (BBL) (Becton, Dickinson and Company, Sparks, MD, USA), as described previously [10]. A macrophage suspension (4 × 10^5^) prepared in Dulbecco’s modified Eagle’s medium (DMEM; Sigma, St. Louis, MO, USA) supplemented with 10% heat-inactivated FBS was added to 96-well tissue culture microplates. The suspension was incubated at 37 °C for 3 h, washed thoroughly to remove nonadherent cells, and incubated again at 37 °C for 24 h. Parasite suspensions in 200 μL of DMEM (2.5 × 10^4^/well, 5 × 10^4^/well, 1 × 10^5^/well) were added to each well. At 2 h post infection, the extracellular parasites were washed away and DMEM supplemented with 10% FBS was added. At 20 h post infection, the culture supernatants (150 μL from each well) were collected for the measurement of cytokines.

### 4.12. Sandwich ELISA for Measuring Cytokine Levels

The level of IFN-γ in the sera and ascites fluid and of IL-6 and IL-12p40 in the macrophage culture supernatant were determined via commercial sandwich ELISAs (Pierce Biotechnology Inc., Rockford, IL, USA), according to the manufacturer’s instructions. The standard cytokine curves constructed from the samples run on the same plate were used for the calculation of cytokine concentrations.

### 4.13. Statistical Analysis

All statistical analyses were performed using GraphPad Prism version 5 (GraphPad Software Inc., La Jolla, CA, USA). Statistical analyses were performed using a one-way analysis of variance (ANOVA) followed by the Tukey–Kramer test for group comparisons in analyzing rates of infection, proliferation, and egress of parasites, and for cytokine production, with the data for each presented as mean values ± standard deviations. The significance of differences in mouse survival was analyzed using a log rank test, and *p-*values < 0.05 were considered statistically significant.

## 5. Conclusions

Based on our previous and current results, the triggered immunity against TgPrx3 antigen during infection might play a role in the host resistance against toxoplasmosis. The disruption of *TgPrx1* or *TgPrx3* did not the hamper the growth of the parasite *in vitro*. On the contrary, survival of mice infected with *TgPrx3*KO was greatly reduced in comparison to those infected with parental and *TgPrx1*KO parasites. Mice infected with *TgPrx1*KO and *TgPrx3*KO showed higher levels of IFN-γ in serum and ascites than the parental-strain-infected group. In addition, higher levels of IL-12 and IL-6 were mostly recorded in *TgPrx3*KO than in parental or *TgPrx1*KO parasites in the infected macrophages *in vitro*, which may have contributed to the adverse effect on mice health and survival. Our current data offer additional evidence for the potential of TgPrx3 as a vaccine candidate against toxoplasmosis.

## Figures and Tables

**Table 1:** Primers used in the current study.

Primer		Sequence (5′-3′)
TgPrx1(II)_236-gRNA	Primer for CRISPR/Cas9 plasmids targeting the *TgPrx3* gene (pSAG1::CAS9-U6::sgTgPrx1)	GCAAGTTCGTGCACAACGCCGTTTTAGAGCTAGAAATAGC
DHFR-25ntTgPrx1(II)_236_1F	To amplify an amplicon containing TgPrx1 homology regions surrounding a pyrimethamine-resistant DHFR* cassette	TGTCGACAGCAAGTTCGTGCACAACAAGCTTCGCCAGGCTGTAAA
DHFR-TgPrx1(II)_236_2R	To amplify an amplicon containing TgPrx1 homology regions surrounding a pyrimethamine-resistant DHFR* cassette	CAATTCCACGTTTCTCCAGGCGGAATTCATCCTGCAAGTGCATAG
TgPrx1(II)_screen_1F (150-169, 20b)	To confirm the insertion of DHFR* cassette into the *TgPrx1* gene	GCCCTTCAGAGATCCTGGCC
TgPrx1(II)_screen_2R (331-350, 20b)	To confirm the insertion of DHFR* cassette into the *TgPrx1* gene	GAACGCCGTAATCTTCTGCC
TgPrx3_289-gRNAv2	Primer for CRISPR/Cas9 plasmids targeting the *TgPrx3* gene (pSAG1::CAS9-U6::sgTgPrx3)	GCCGATGCCGTCATGCCGAAGTTTTAGAGCTAGAAATAGC
DHFR-25ntTgPrx3(II)_289_1F	To amplify an amplicon containing TgPrx3 homology regions surrounding a pyrimethamine-resistant DHFR* cassette	ACTTCACAGCCGATGCCGTCATGCCAAGCTTCGCCAGGCTGTAAA
DHFR-TgPrx3(II)_289_2Rv2	To amplify an amplicon containing TgPrx3 homology regions surrounding a pyrimethamine-resistant DHFR* cassette	AGTTTCTGAATCTCACCGTTCGGAATTCATCCTGCAAGTGCATAG
TgPrx3(II)_screen_1F (240-259, 20b)	To confirm the insertion of DHFR* cassette into the *TgPrx3* gene	CAGTCCGAACGGACTCTGCC
TgPrx3(II)_screen_2R (360-379, 20b)	To confirm the insertion of DHFR* cassette into the *TgPrx3* gene	GATAAAACAGCAGAACGACG

## Data Availability

The data of this research can be provided based on request from the correspondence author (project supervisor).

## Supplementary Materials

The following supporting information can be downloaded at https://www.mdpi.com/article/10.3390/ijms23063076/s1.

## References

[1] Dubey J.P. (2010). Toxoplasmosis of Animals and Humans.

[2] Tenter A.M., Heckeroth A.R., Weiss L.M. (2000). *Toxoplasma gondii*: From animals to humans. Int. J. Parasitol..

[3] Hakimi M.A., Olias P., Sibley L.D. (2017). *Toxoplasma* effectors targeting host signaling and transcription. Clin. Microbiol. Rev..

[4] Kwok L.Y., Schluter D., Clayton C., Soldati D. (2004). The antioxidant systems in *Toxoplasma gondii* and the role of cytosolic catalase in defence against oxidative injury. Mol. Microbiol..

[5] Son E.S., Song K.J., Shin J.C., Nam H.W. (2001). Molecular cloning and characterization of peroxiredoxin from *Toxoplasma gondii*. Korean J. Parasitol..

[6] Akerman S.E., Müller S. (2005). Peroxiredoxin-linked detoxification of hydroperoxides in *Toxoplasma gondii*. J. Bio. Chem..

[7] Sautel C.F., Ortet P., Saksouk N., Kieffer S., Garin J., Bastien O., Hakimi M.A. (2009). The histone methylase KMTox interacts with the redox-sensor peroxiredoxin-1 and targets genes involved in *Toxoplasma gondii* antioxidant defences. Mol. Microbiol..

[8] Marshall E.S., Elshekiha H.M., Hakimi M.A., Flynn R.J. (2011). *Toxoplasma gondii* peroxiredoxin promotes altered macrophage function, caspase-1-dependent IL-1β secretion enhances parasite replication. Vet. Res..

[9] Fereig R.M., Nishikawa Y. (2016). Peroxiredoxin 3 promotes IL-12 production from macrophages and partially protects mice against infection with *Toxoplasma gondii*. Parasitol. Int..

[10] Fereig R.M., Kuroda Y., Terkawi M.A., Mahmoud M.E., Nishikawa Y. (2017). Immunization with *Toxoplasma gondii* peroxiredoxin 1 induces protective immunity against toxoplasmosis in mice. PLoS ONE.

[11] Deponte M., Becker K. (2005). Biochemical characterization of *Toxoplasma gondii* 1-Cys peroxiredoxin 2 with mechanistic similarities to typical 2-Cys Prx. Mol. Biochem. Parasitol..

[12] Suarez C.E., Bishop R.P., Alzan H.F., Poole W.A., Cooke B.M. (2017). Advances in the application of genetic manipulation methods to apicomplexan parasites. Int. J. Parasitol..

[13] Shen B., Brown K.M., Lee T.D., Sibley L.D. (2014). Efficient gene disruption in diverse strains of *Toxoplasma gondii* using CRISPR/CAS9. mBio.

[14] Sidik S.M., Hackett C.G., Tran F., Westwood N.J., Lourido S. (2014). Efficient genome engineering of *Toxoplasma gondii* using CRISPR/CAS9. PLoS ONE.

[15] Ihara F., Fereig R.M., Himori Y., Kameyama K., Umeda K., Tanaka S., Ikeda R., Yamamoto M., Nishikawa Y. (2020). *Toxoplasma gondii* dense granule proteins 7, 14, and 15 are involved in modification and control of the immune response mediated via NF-κB pathway. Front. Immunol..

[16] Angelucci F., Miele A.E., Ardini M., Boumis G., Saccoccia F., Bellelli A. (2016). Typical 2-Cys peroxiredoxins in human parasites: Several physiological roles for a potential chemotherapy target. Mol. Biochem. Parasitol..

[17] Haraldsen J.D., Liu G., Botting C.H., Walton J.G., Storm J., Phalen T.J., Kwok L.Y., Soldati-Favre D., Heintz N.H., Müller S. (2009). Identification of Conoidin a as a covalent inhibitor of Peroxiredoxin II. Org. Biomol. Chem..

[18] Rosowski E.E., Lu D., Julien L., Rodda L., Gaiser R.A., Jensen K.D., Saeij J.P. (2011). Strain-specific activation of the NF-kappaB pathway by GRA15, a novel *Toxoplasma gondii* dense granule protein. J. Exp. Med..

[19] Li J., Guo H., Galon E.M., Gao Y., Lee S.H., Liu M., Li Y., Ji S., Jia H., Xuan X. (2020). Hydroxylamine and Carboxymethoxylamine can inhibit *Toxoplasma gondii* growth through an aspartate aminotransferase-independent oathway. Antimicrob. Agents Chemother..

[20] Gazzinelli R.T., Hakim F.T., Hieny S., Shearer G.M., Sher A. (1991). Synergistic role of CD4+ and CD8+ T lymphocytes in IFN-gamma production and protective immunity induced by an attenuated *Toxoplasma gondii* vaccine. J. Immunol..

[21] Sibley L.D., Adam L., Fukutomi Y., Krahenbuhl J.L. (1991). Tumor necrosis factor-alpha triggers antitoxoplasmal activity by IFN-gamma primed macrophages. J. Immunol..

[22] Gazzinelli R.T., Hieny S., Wynn T.A., Wolf S., Sher A. (1993). Interleukin 12 is required for the T-lymphocyte-independent induction of interferon-γ by an intracellular parasite and induces resistance in T-cell deficient hosts. Proc. Natl. Acad. Sci. USA.

[23] Kono K., Salazar-Onfray F., Petersson M., Hansson J., Masucci G., Wasserman K., Nakazawa T., Anderson P., Kiessling R. (1996). Hydrogen peroxide secreted by tumor-derived macrophages down-modulates signal-transducing zeta molecules and inhibits tumor-specific T cell-and natural killer cell-mediated cytotoxicity. Eur. J. Immunol..

[24] Nathan C., Shiloh M.U. (2000). Reactive oxygen and nitrogen intermediates in the relationship between mammalian hosts and microbial pathogens. Proc. Natl. Acad. Sci. USA.

[25] Nishikawa Y., Shimoda N., Fereig R.M., Moritaka T., Umeda K., Nishimura M., Ihara F., Kobayashi K., Himori Y., Suzuki Y. (2018). *Neospora caninum* dense granule protein 7 regulates the pathogenesis of neosporosis by modulating host immune response. Appl. Environ. Microbiol..

