# Supplementary material for: Genetic Disruption of Toxoplasma gondii peroxiredoxin (TgPrx) 1 and 3 Reveals the Essential Role of TgPrx3 in Protecting Mice from Fatal Consequences of Toxoplasmosis
Source: Int J Mol Sci. 2022 Mar 12;23(6):3076. doi: 10.3390/ijms23063076 (PMC8951120; doi:10.3390/ijms23063076)
Supplement: Supplementary file 1 [file ijms-23-03076-s001.zip › ijms-1595570-supplementary.pdf]

Supplemental Information

Materials and methods

1. Plasmid construction

A plasmid expressing CAS9 and a single guide RNA (sgRNA) targeting the UPRT gene of *T. gondii* (pSAG1::CAS9U6::sgUPRT) were obtained from Addgene (Cambridge, MA, USA). The sgRNAs were designed based with EuPaGDT (http://grna.ctegd.uga.edu). The CRISPR/CAS9 plasmids (pSAG1::CAS9-U6::sgTgPrx1 ans pSAG1::CAS9-U6::sgTgPrx3) were generated with the Q5® Site-Directed Mutagenesis Kit (New England Biolabs, Ipswich, MA, USA) from pSAG1::CAS9-U6::sgUPRT, changing the UPRT-targeting gRNA to other specific sgRNAs using the primers listed in Table 1. More details for plasmids used in the current study are listed in Table S1.

Table S1: Plasmids used in the current study.

Plasmid	Description	Use	Source or reference
pSAG1:CAS9-U6:sgUPRT	CAS9 expressed from the *Toxoplasma* SAG1 promoter and CRISPR gRNA targeting *Toxoplasma* UPRT produced from the U6 promoter	CRISPR plasmid targeting *Toxoplasma* UPRT	Addgene
pSAG1:CAS9-U6:sgTgPrx1	CAS9 expressed from the *Toxoplasma* SAG1 promoter and CRISPR gRNA targeting TgPrx1 produced from the U6 promoter	CRISPR plasmid targeting between nucleotides 236 and 237 in TgPrx1 gene	This study
pSAG1:CAS9-U6:sgTgPrx3	CAS9 expressed from the *Toxoplasma* SAG1 promoter and CRISPR gRNA targeting TgPrx3 produced from the U6 promoter	CRISPR plasmid targeting between nucleotides 289 and 290 in TgPrx3 gene	This study
pUPRT-DHFR-D	DHFR* cassette flanked by two homology arms from the 5′- and 3′-UTR of UPRT gene respectively	Replacing the UPRT gene with DHFR*	Addgene
pGEX-4T3	pGEX4T-3 plasmid	Preparation of recombinant protein of glutathione Stransferase (GST)	Amersham
pGEX-4T3-TgPrx1	Cloned TgPrx1 gene into pGEX4T-3 plasmid	Preparation of recombinant protein of TgPrx1 fused with GST	Fereig et al., 2017
pGEX-4T3-TgPrx3	Cloned TgPrx3 gene into pGEX4T-3 plasmid	Preparation of recombinant protein of TgPrx3 fused with GST	Fereig and Nishikawa, 2016

2. Clinical scores and body weights

Clinical findings of infected female ICR with PruΔku80Δhxgprt, *TgPrx1* deficient parasite clone 1 (*TgPrx1*KO1) and *TgPrx3* deficient parasite clone 2 (*TgPrx3*KO2) were monitored daily to from −2 to 30 dpi. The clinical score was adjusted by recording the clinical signs manifested in each mouse and the mouse group overall starting from −2 to 30 dpi with different parasite lines as described in our previous study **(Abe et al., 2015)**. Briefly, each recorded clinical sign was represented by a score ranging from 0 (no signs) to 10 (all signs) (Figure S3A). In addition, the weights of the individual mice on the first day of measurement were compared between different Groups (Figure S3B).

3. DNA isolation and real-time PCR analysis of parasite burden in brain

DNA was extracted from the brain tissues and processed as described previously **(Fereig et al., 2017)**. Each organ was thawed in 10 volumes of extraction buffer (0.1 M Tris-HCl [pH 9.0], 1% SDS, 0.1 M NaCl, 1 mM EDTA) and 100 µg/ml proteinase K at 55°C. The DNA was purified with phenol-chloroform extraction and ethanol precipitation. The parasite DNA was then amplified with primers specific to the *T. gondii* B1 gene. Amplification, data acquisition, and data analysis were performed in the ABI Prism 7900HT sequence detection system (Applied Biosystems), and the cycle threshold values (CT) were calculated as described previously (10, 38). A standard curve was constructed using 10-fold serial dilutions of *T. gondii* DNA extracted from 10^5 parasites; thus, the curve ranged from 0.01 to 10,000 parasites. The parasite number was calculated from the standard curve.

4. Indirect ELISA

An amount of 50 μL of recombinant antigens TgGRA7 at a final concentration of 0.1 μM were coated onto ELISA plates (Nunc, Roskilde, Denmark), and were incubated overnight at 4 °C, and performed as described previously **(Terkawi et al., 2013)**. Antigens were diluted in 0.05 M carbonate buffer (pH 9.6). The plates were washed once with washing buffer (0.05% Tween 20 in PBS) and then blocked with PBS containing 3% skimmed milk (PBS-SM) for 1 h at 37 °C and treated against the test sample. An amount 50 μl of serum samples (diluted with PBS-SM at 1:200) were placed to the wells. After the plates were washed six times, plates were then incubated at 37 °C for 1 h with horseradishperoxidase-conjugated anti-mouse IgG (Bethyl Laboratories, Montgomery, TX, USA), diluted with PBS-SM at 1:4000). The plates were washed six times, and 100 μL of substrate solution (0.1 M citric acid, 0.2 M sodium phosphate, 0.003% H_2O_2, 0.3 mg/mL 2,2′-azinobis [3-ethylbenzothiazoline-6 sulphonic acid]; Sigma) was added to each well and kept at room temperature in the dark for 1 h. The absorbance was measured using an ELISA reader at a wavelength of 415 nm was determined with a plate reader (Corona Microplate Reader MTP-120; Corona, Tokyo, Japan).

5. Statistical analysis

The significance in change in body weight or clinical score was determined by two-way ANOVA plus Bonferroni post hoc analysis ($P < 0.05$). Quantification of parasite

burden in the brains and specific antibody response against TgGRA7 was analyzed with a one-way ANOVA plus a Tukey–Kramer post hoc analysis $P < 0.05$. All statistical analyses were performed with GraphPad Prism version 5 (GraphPad Software Inc., La Jolla, CA, USA).

Supplemental references

Abe, C., Tanaka, S., Nishimura, M., Ihara, F., Xuan, X., Nishikawa, Y., 2015. Role of the chemokine receptor CCR5-dependent host defense system in Neospora caninum infections. Parasites Vectors. https://doi.org/10.1186/s13071-014-0620-5.

Fereig, R. M., Kuroda, Y., Terkawi, M. A., Mahmoud, M. E., & Nishikawa, Y. (2017). Immunization with Toxoplasma gondii peroxiredoxin 1 induces protective immunity against toxoplasmosis in mice. *PloS one*, *12*(4), e0176324. https://doi.org/10.1371/journal.pone.0176324

Fereig, R.M.; Nishikawa, Y. Peroxiredoxin 3 promotes IL-12 production from macrophages and partially protects mice against infection with *Toxoplasma gondii*. *Parasitol. Int.* **2016**, *65*, 741–748. https://doi.org/10.1016/j.parint.2016.09.008

Terkawi, M.A.; Kameyama, K.; Rasul, N.H.; Xuan, X.; Nishikawa, Y. Development of an immunochromatographic assay based on dense granule protein 7 for serological detection of *Toxoplasma gondii* infection. *Clin. Vaccine Immunol.* **2013**, *20*, 596–601. https://doi.org/10.1128/CVI.00747-12.

Supplemental figures

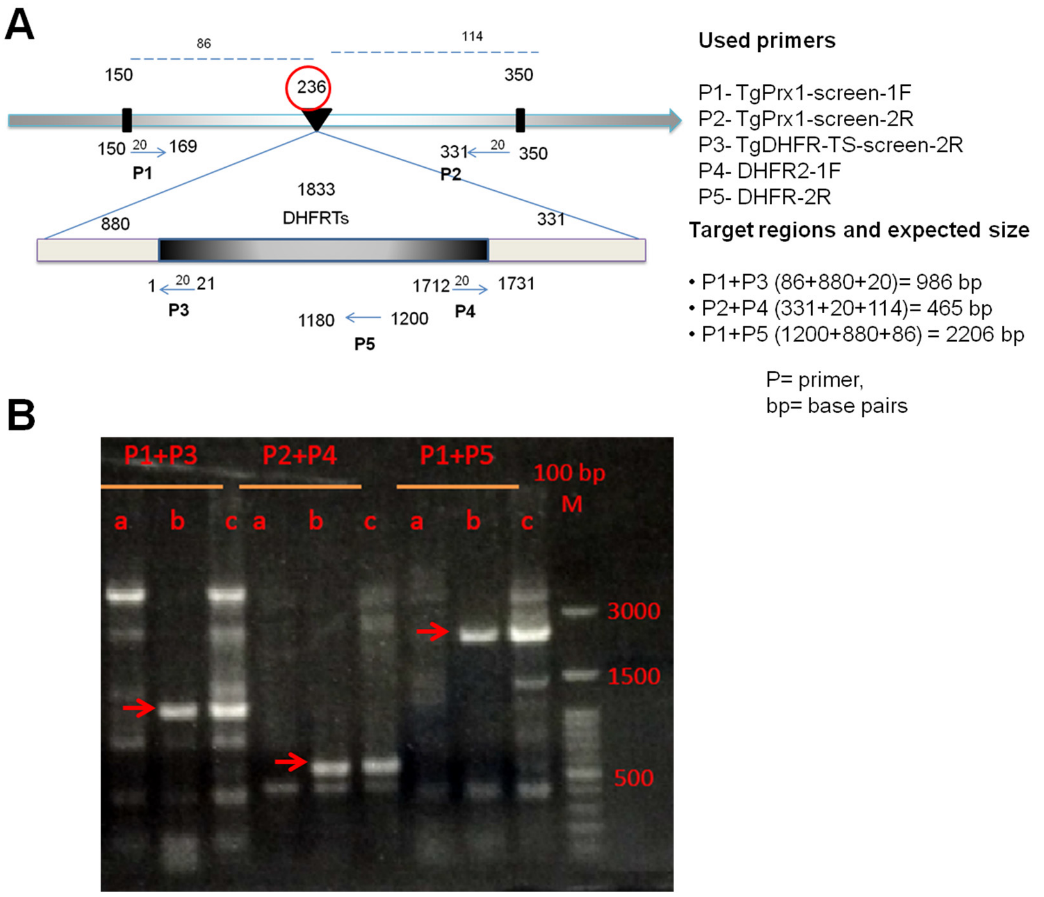

Figure S1. Generation of *TgPrx1*KO parasites using CRISPR/CAS9. **(A)** Schematic representation of the CRISPR/CAS9 strategy used to inactivate the target genes by inserting the pyrimethamine-resistance DHFR cassette (DHFR∗). Red circle refers to guide RNA of *TgPrx1* targeted for plasmid insertion. Transfection of the CRISPR plasmid targeting *TgPrx1*, together with an amplicon containing the DHFR∗-expressing cassette flanked by regions homologous to the target gene, was used to disrupt the corresponding target gene by insertion. Different primers including sequences from the target locus of *TgPrx1* and DHFR cassette were used to confirm the successful gene disruption. **(B)** PCR confirmation of *TgPrx1* disruption. Genomic DNA from parental parasite PruΔku80Δhxgprt, knockout clones *TgPrx1*KO1 and *TgPrx1*KO2 was used for PCR amplification using the primer set described at panel **A**. P refers to primer, a; PruΔku80Δhxgprt, b; *TgPrx1*KO clone 1, c; *TgPrx1*KO clone 2. Red arrows indicate the positive bands at expected sizes.

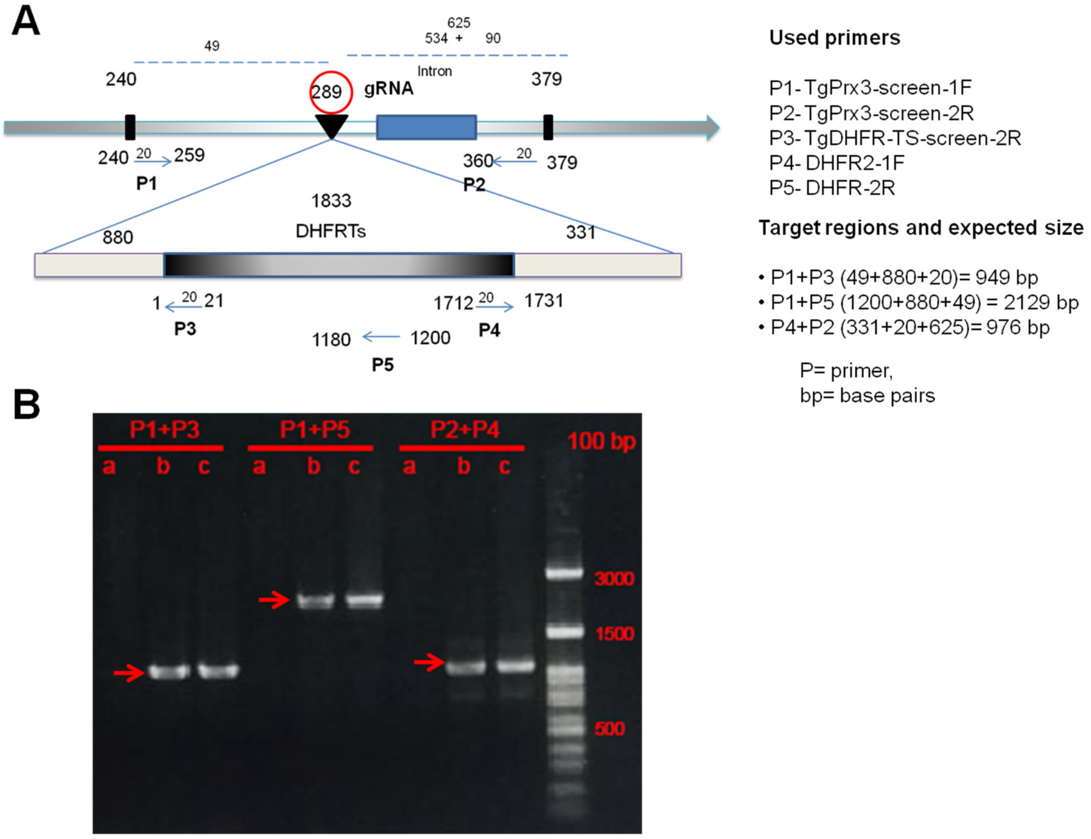

Figure S2: Generation of *TgPrx*3KO parasites using CRISPR/CAS9. **(A)** Schematic representation of the CRISPR/CAS9 strategy used to disrupt the target genes by inserting the pyrimethamine-resistance DHFR cassette (DHFR∗). Red circle refers to guide RNA of *TgPrx3* targeted for plasmid insertion. Transfection of the CRISPR plasmid targeting *TgPrx3*, together with an amplicon containing the DHFR∗-expressing cassette flanked by regions homologous to the target gene, was used to disrupt the corresponding target gene by insertion. **(B)** PCR confirmation of *TgPrx3* disruption. Genomic DNA from parental parasite PruΔku80Δhxgprt *(TgPru)*, knockout clones *TgPrx*3KO2 and *TgPrx*3KO5 was used for PCR amplification using the primer set described at panel (**A**). P; refers to primer, a; PruΔku80Δhxgprt, b; *TgPrx*3KO clone 2, c; *TgPrx*3KO clone 5. Red arrows indicate the positive bands at expected sizes.

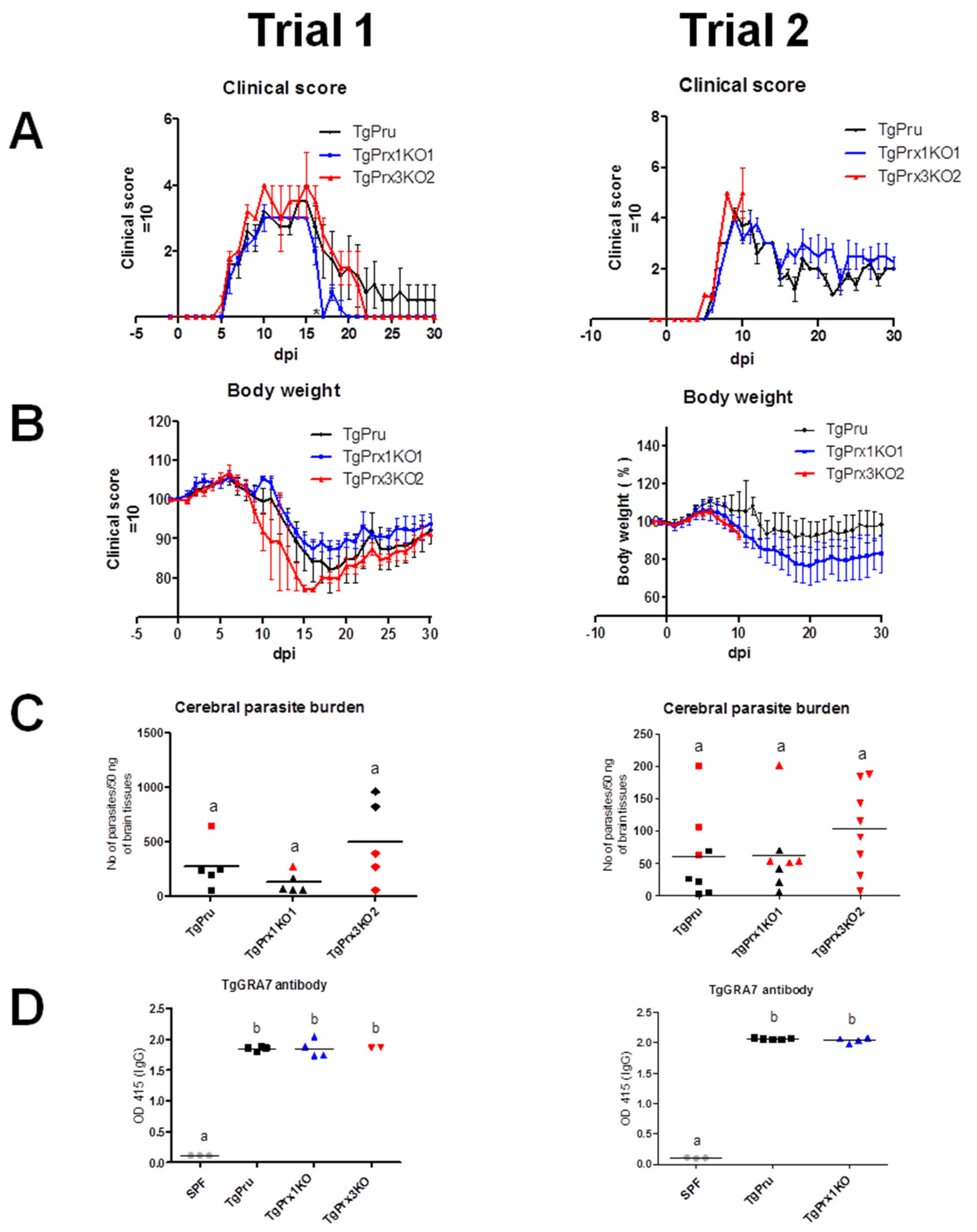

Figure S3: Clinical findings, parasite burden and antibody response. Female ICR mice were infected intraperitoneally with ($5x10^4$) tachyzoites of PruΔku80Δhxgprt (TgPru), *TgPrx1* deficient parasite clone 1 (*TgPrx1*KO1) or *TgPrx3* deficient parasite clone 2 (*TgPrx3*KO2) (n = 5 for trial 1 and n = 8 for trial 2). Changes in the clinical score (A) and body weight (B) were calculated as the means ± standard deviation of clinical score and body weight values of all mice in a group from 2 to +30 dpi. The significance in change in body weight or

clinical score was determined by two-way ANOVA plus Bonferroni post hoc analysis ($P < 0.05$). Asterisk (*) refers to a significant difference in the test groups. (C) Quantified parasite DNA in the brains of the surviving mice at 30 dpi (Trial 1; TgPru 4/5, *TgPrx1*KO1 4/5, *TgPrx3*KO2 2/5, trial 2; TgPru 5/8, *TgPrx1*KO1 4/8). The results were analyzed with a one-way ANOVA plus a Tukey–Kramer post hoc analysis, but there were no significant differences. Red symbols refer to samples collected from freshly dead mice. (D) Specific antibody response against TgGRA7 in female ICR mice. Sera were collected from all surviving mice in each group at the end of experiment 30 dpi (Trial 1; TgPru 4/5, *TgPrx1*KO1 4/5, *TgPrx3*KO2 2/5, trial 2; TgPru 5/8, *TgPrx1*KO1 4/8). In addition, sera from female ICR before infection (SPF; specific pathogen free) were collected and used as negative controls ($n = 3$). The mean optical density (OD) was determined at a wavelength of 415 nm. Each bar represents the mean ± standard deviation for mice in each group. The different letters above the bars in the graphs indicate statistically significant differences among the different test groups (one-way ANOVA plus Tukey–Kramer post hoc analysis, $P < 0.05$).
